# TMD-Unet: Triple-Unet with Multi-Scale Input Features and Dense Skip Connection for Medical Image Segmentation

**DOI:** 10.3390/healthcare9010054

**Published:** 2021-01-06

**Authors:** Song-Toan Tran, Ching-Hwa Cheng, Thanh-Tuan Nguyen, Minh-Hai Le, Don-Gey Liu

**Affiliations:** 1 Program of Electrical and Communications Engineering, Feng Chia University, Taichung 40724, Taiwan; nttuan@kgc.edu.vn (T.-T.N.); lmhai@tvu.edu.vn (M.-H.L.); dgliu@fcu.edu.tw (D.-G.L.); 2 Department of Electrical and Electronics, Tra Vinh University, Tra Vinh 87000, Vietnam; 3 Department of Electronic Engineering, Feng Chia University, Taichung 40724, Taiwan; chengch@fcu.edu.tw

**Keywords:** medical image segmentation, nuclei segmentation, liver segmentation, polyp segmentation, skin lesion segmentation, spleen segmentation, left atrium segmentation, electron microscopy segmentation, Unet architecture

## Abstract

Deep learning is one of the most effective approaches to medical image processing applications. Network models are being studied more and more for medical image segmentation challenges. The encoder–decoder structure is achieving great success, in particular the Unet architecture, which is used as a baseline architecture for the medical image segmentation networks. Traditional Unet and Unet-based networks still have a limitation that is not able to fully exploit the output features of the convolutional units in the node. In this study, we proposed a new network model named TMD-Unet, which had three main enhancements in comparison with Unet: (1) modifying the interconnection of the network node, (2) using dilated convolution instead of the standard convolution, and (3) integrating the multi-scale input features on the input side of the model and applying a dense skip connection instead of a regular skip connection. Our experiments were performed on seven datasets, including many different medical image modalities such as colonoscopy, electron microscopy (EM), dermoscopy, computed tomography (CT), and magnetic resonance imaging (MRI). The segmentation applications implemented in the paper include EM, nuclei, polyp, skin lesion, left atrium, spleen, and liver segmentation. The dice score of our proposed models achieved 96.43% for liver segmentation, 95.51% for spleen segmentation, 92.65% for polyp segmentation, 94.11% for EM segmentation, 92.49% for nuclei segmentation, 91.81% for left atrium segmentation, and 87.27% for skin lesion segmentation. The experimental results showed that the proposed model was superior to the popular models for all seven applications, which demonstrates the high generality of the proposed model.

## 1. Introduction

Presently, medical image types of equipment are evolving and popular such as computed tomography (CT), magnetic resonance imaging (MRI), X-ray, and ultrasound. Medical imaging analysis plays an important role in facilitating faster and more accurate diagnosis and treatment. Medical image segmentation is one of the most concerning challenges in recent years [1]. Although many published approaches have been achieved with certain successes, medical image segmentation is still a challenging topic due to the difficulty of feature analysis [2]. It is difficult to extract the features because the medical image is often low in contrast, blurred, and noisy. There are many approaches to medical image analysis; however, deep learning has been showing remarkable improvement in recent years [3]. With deep learning, extracting and analyzing image features will be done easier and faster, thereby significantly improving the results of image segmentation. The number of published studies has increased dramatically each year. Specifically, the number of articles using deep learning for medical image processing published in 2018 was 100 times more than in 2014 [4].

The purpose of medical image segmentation is to classify the pixels in an image, thereby identifying internal organs, recognizing abnormal areas such as tumors, lesions, etc. To accomplish this goal, deep learning researchers have proposed an encoder–decoder structure such as fully convolution network (FCN) [5], Deeplab [6], Unet [7], etc. These network models are applicable for medical image segmentation applications such as liver and liver tumor [8,9,10], brain and brain tumor [11,12,13], lung and lung nodule [14,15], nuclei [16,17], polyp [18,19], skin lesion [20,21,22], etc. Many studies have proposed these models for many different types of medical imaging [23,24,25,26,27].

The Unet model is one of the most successful architectures in medical image segmentation challenges [28]. The advantages of Unet are encoder–decoder structure and skip connection. The encoder block is used to extract image features while the decoder block is used to recover the image to original size from the extracted features and to output the final result of the segmentation. The skip connection combines low-level features in the encoder block with the high-level features in the decoder block. The coarse-grained features are also concatenated with fine-grained features by the skip connection. Although there are many outstanding advantages, the Unet model still has some limitations such as the structure of the model is not flexible when training with different size datasets, and the skip connection has not fully exploited the features from the encoder block. Due to the dominance of Unet, recent studies have focused on further improving the structure of Unet for application on medical image segmentation. The approaches of these studies were to change the internal structure of the nodes in the encoder and decoder blocks [29,30,31,32] or change the connection between the blocks [33,34]. Other approaches were to change the skip connection of the conventional Unet architecture [9,35,36,37]. Some studies used a cascade structure [10,38,39], or used the hybrid methods [8,40,41,42].

The standard convolution in the nodes of the Unet model is still quite simple. Therefore, some studies have focused on improving the efficiency of feature extraction from nodes by replacing the structure of the node or proposed a new convolutional function. Chen et al. [29] proposed a spatial channel-wise convolution to extract the features from the relationship between the spatial information of the pixels. They introduced an end-to-end network based on Unet structure by adding the new convolution in the encoder and decoder nodes. To improve the efficiency of the standard convolution, Chen et al. [30] proposed a new network structure named DRI-Net, which combined the advantages of three popular network structures: densenet [43], inception [44], and residual [45] in a node of the network.

Skip connection path is also extremely interested because it is an outstanding advantage over other network models. Improving skip connection performance will lead to an increase in the efficiency of the entire model. Huang et al. [33] used the dense skip connections to combine all of the features from the encoder node with the features from the decoder node. The full-scale aggregated feature maps are learned by deep supervision. Zang et al. [34] changed the connection between the nodes in the encoder and decoder block in the traditional Unet. They also applied the dense skip connection from the encoder node to the decoder node. Zhou et al. [35] introduced the Unet++ model, which exploited the multi-scale features by using the nested skip connection. The skip connections included the convolution units that are connected as a dense network. Ibtehaz and Rahman [36] proposed MultiResUnet to improve the convolution structure in the node of the conventional Unet. They introduced the multiRes block, which used multiple 3 × 3 filters to replace the 3 × 3, 5 × 5, and 7 × 7 filters arranged in parallel. The skip connection path, which consists of 3 × 3 filters and 1 × 1 filters accompany the residual connections replaced the traditional skip connection. Liu et al. [37] integrated the multi-scale input, multi-scale side output, and attention mechanism into the Unet++ for optical coherence tomography image segmentation. 

The cascade network structure in the medical image segmentation has the advantage of reducing the number of false positives. The hybrid architecture will increase the efficiency of feature extraction. Many studies have used cascade or hybrid structure to improve segmentation efficiency. Xi et al. [10] used the Unet models in cascade structure for liver and liver tumor segmentation. They used the first model for liver segmentation and the second one to segment the liver tumor. Jiang et al. [41] joint the soft and hard attention mechanisms. The long and the short skip connections were combined. For liver tumor segmentation, they also applied a cascade structure. Li et al. [8] proposed the hybrid network named H-DenseUnet to combined a 2D and a 3D model. The 2D model extracted the intra-slice features in 2D images while the 3D model aggregated the volumetric contexts, and then used a hybrid feature fusion layer to combine and optimize the 2D and 3D features. However, H-DenseUnet is complex and consumes a large memory because of the number of parameters. To improve this problem, Zhang et al. [42] proposed the light-weight hybrid convolution network, which used the depthwise and spatiotemporal separate block and the separable convolution. This model is a similar structure, but the number of parameters and the calculation time is less than H-DenseUnet.

**Figure 1:**
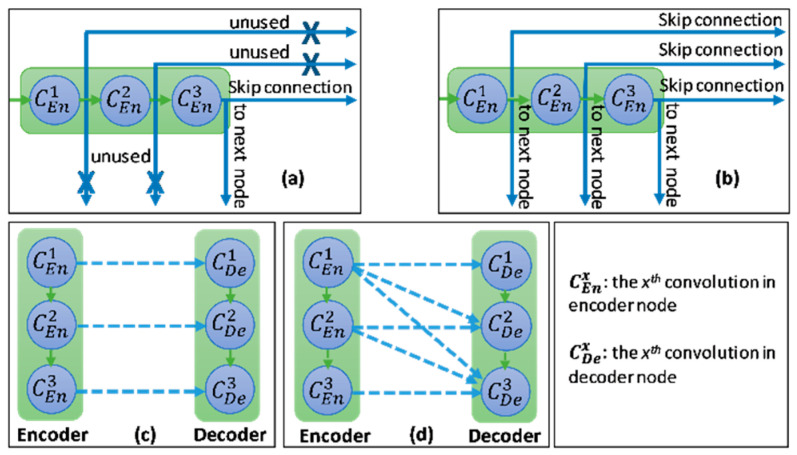
Illustration of the divergences between the connection of (**a**) tradition Unet and (**b**) the proposed model; the difference between (**c**) skip connection and (**d**) dense skip connection. The blue line denotes the output feature map of the convolution unit while the blue dashed arrow depicts the skip connection path; C(x,en): the xth convolution in encoder node; C(x,de): the xth convolution in decoder node.

In this study, we introduce a new network architecture named TMD-Unet. Inspired by the hybrid and cascade architecture, we modified the node structures of the traditional Unet model. Each node was composed of three convolution units. Most Unet-based models ignored the output feature maps in nodes, only the last output in the node were used. Furthermore, for cascade architecture models, the outputs of the encoder node were not reused for the next layer. In the proposed model, the output features of convolution units would be used as skip connections and input for the next nodes. The new network included three sub-Unet models arranged in parallel. The skip connection also plays an important role in the Unet architecture. In this study, we applied a dense skip connection (DS) to enhance the efficiency of low-level features from the encoder block. Figure 1 presents the differences between the connection of conventional Unet and the proposed model, the skip connection in cascade structure and the DS. The multi-scale input (MSI) was also integrated in the proposed model. The advantage of the MSI is the fusion of the input images information with many different scales, thereby, enhancing the input features of the model.

The main contributions of the paper are summarized as follows:

1. We introduce a new deep learning model named TMD-Unet, for medical image segmentation. The TMD-Unet, which included three sub-Unet models, exploited the output features of convolutional units effectively.
2. We found that integrating the DS and MSI into the model improved the performance of the network. The number of parameters changed slightly when applying DS and MSI; however, the model performance got much improvement.
3. The evaluations were performed on seven datasets. We demonstrated the applicability of TMD-Unet for a variety of medical imaging including MRI, CT, dermoscopy, colonoscopy, and electron microscopy.

## 2. Proposed Method 

In this section, the structure of the proposed network is described in detail. The connection details in the model are also explained clearly and transparently. In this study, we first proposed the Triple-Unet (T-Unet) model. The T-Unet model is based on the traditional Unet with three main changes: (i) modifying the node structure, each node consists of three densely connected convolution units based on a dense structure [43]; (ii) exploiting all output features of unit convolution; (iii) using dilated convolution (DC) [46] instead of standard convolution. The TMD-Unet model is further developed by integrating DS and MSI into the T-Unet model. 

### 2.1. Triple-Unet (T-Unet) Structure and Multi-Scale Input Features (MSI)

**Figure 2:**
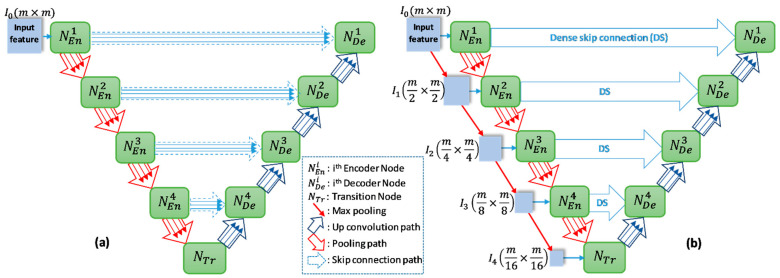
The network structures of (**a**) T-Unet and (**b**) TMD-Unet. There are five input features in the TMD-Unet model. “*mxm*” indicates the 2D size of the input features.

**Figure 3:**
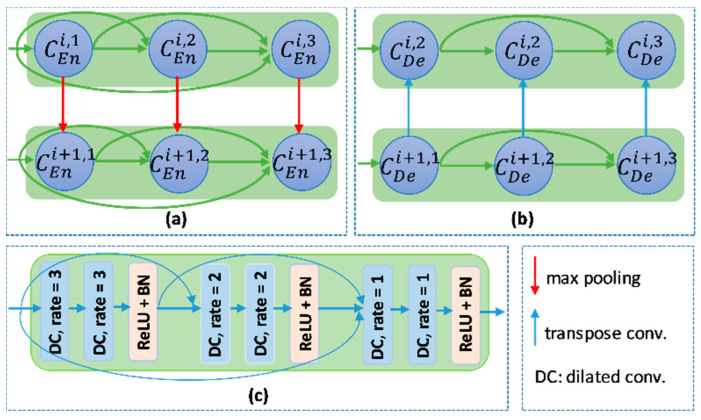
The connection between two nodes in (**a**) the encoder part and (**b**) the decoder part. (**c**) the detail of the encoder node.

The overview of proposed networks is presented Figure 2. Inspired by conventional Unet, the proposed models also consist of two main blocks: encoder and decoder. Each block includes four nodes. In addition, a transition node is at the bottom of the network. The node of T-Unet model included three convolution units, which consist of two 3 × 3 convolutions followed by a ReLU activation and batch normalization (BN) (Figure 3c). Let $I_{0}\in R^{m\times m\times n}$ is an input feature of the model after applied the convolution for the input tensor, where *n* is the number of filters and *m* × *m* indicates the size of the input image. In the encoder part, the output features of the first node described as:

(1) $$\chi_{1(T)}^{i}=\chi_{1(TMD)}^{i}={\mathbb{C}}^{4-i}\left(\left[{\left[\chi_{1}^{k}\right]}_{k=1}^{i-1},I_{0}\right]\right),\;\;with\;\;i=[1,3]$$

where $\chi_{1(T)}^{i}$ and $\chi_{1(TMD)}^{i}$ are the *i^th^* output feature of the first encoder node of T-Unet and TMD-Unet, respectively, with $\chi_{1}^{0}=\emptyset$. ${\mathbb{C}}^{r}$ denotes the dilated convolution with dilation rate equal to *r*, and [.] defines the concatenate function. Figure 3a,b describer the connection between two nodes in detail. 

From the second node, the inputs of T-Unet are the features after pooled from the previous nodes and the features come from the previous convolution unit while the input of TMD-Unet also has added the scaled input features. To create the input features for the encoder node, we simply applied the max-pooling function, the size of the input features will be halved after pooled. The expressions to represent the input features and outputs are as follows:

(2) $$I_{j}=M\left(I_{j-1}\right),\;\;with\;\;j=\left[1,4\right]$$

(3) $$\chi_{h(T)}^{i}={\mathbb{C}}^{4-i}\left(\left[{\left[\chi_{h}^{k}\right]}_{k=1}^{i-1},M\left(\chi_{h-1}^{i}\right)\right]\right)$$

(4) $$\chi_{h(TMD)}^{i}={\mathbb{C}}^{4-i}\left(\left[{\left[\chi_{h}^{k}\right]}_{k=1}^{i-1},M\left(\chi_{h-1}^{i}\right),I_{h}\right]\right)$$

where *h* ϵ [2,5] indicates the order of the encoder node with $\chi_{5}$ is the output features of the transition node, *M(.)* is the max-pooling function. By exploiting the feature maps of each convolution unit in the node, the proposed networks compose of three sub-Unet models that are arranged in parallel. The architecture of the sub-Unet is connected as

$$\left\{C_{En}^{i,1}\to C_{En}^{i,2}\to C_{En}^{i,3}\to C_{En}^{i,4}\to C_{Tr}^{i}\to C_{De}^{i,4}\to C_{De}^{i,3}\to C_{De}^{i,2}\to C_{De}^{i,1}\right\}$$

where i=(1,2,3), and $C_{Tr}^{i}$ is the convolution unit of the transition node.

### 2.2. Dense Skip Connection (DS)

**Figure 4:**
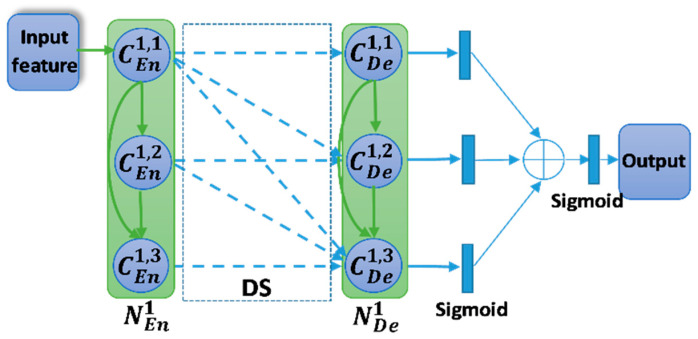
The dense skip connection between the encoder node and decoder node. The blue dashed arrow denotes the skip connection. Symbol ⊕ indicates the concatenation operator.

In the conventional Unet, the skip connection is the output feature from the encoder node. It is only the output of the last convolution unit. In the T-Unet, all the output features in the encoder node are used as the skip connection. Figure 4 describes the dense skip connection between the first encoder node and the first decoder node. The detail of the final output is also presented in Figure 4. The inputs of each convolution unit in the decoder node composed of the features from the lower node, from the previous convolution units, and the skip connection. Let $\gamma_{h}^{i}$ is the output feature of *i^th^* convolution unit in the *h^th^* decoder node. The calculation formula is defined as:

(5) $$\gamma_{h(T)}^{i}={\mathbb{C}}^{4-i}\left(\left[{\left[\gamma_{h}^{k}\right]}_{k=1}^{i-1},\tau \left(\gamma_{h+1}^{i}\right),\chi_{h}^{i}\right]\right),\;\;i\in \left[1;3\right]$$

(6) $$\gamma_{h(TMD)}^{i}={\mathbb{C}}^{4-i}\left(\left[{\left[\gamma_{h}^{k}\right]}_{k=1}^{i-1},\tau \left(\gamma_{h+1}^{i}\right),{\left[\chi_{h}^{m}\right]}_{m=1}^{i}\right]\right),\;\;i\in \left[1;3\right]$$

where τ(·) is the transposed convolution. $\gamma_{h(T)}^{i}$ and $\gamma_{h(TMD)}^{i}$ are the output feature of *i^th^* convolution unit in the *h^th^* decoder node of T-Unet and TMD-Unet, respectively.

There are three outputs from three sub-Unet models. They will be applied a 1 × 1 convolution and a sigmoid function and then be concatenated. The final output is also obtained by the 1 × 1 convolution and sigmoid function, and it is described as follow,

(7) $$F=\delta \left(\Gamma \left({\left[\delta \left(\Gamma \left(\gamma_{1}^{i}\right)\right)\right]}_{i=1}^{3}\right)\right)$$

where *F* is the final output, δ(·) indicates the sigmoid function, and Γ(·) is the 1 × 1 convolution.

Table 1 describes the architecture of the TMD-Unet model. The size of the convolution in the model is 3 × 3. All the convolution layers applied a dropout rate of 0.2.

## 3. Experiments 

### 3.1. Datasets and Pre-Processing

To demonstrate the effectiveness and generality of the proposed model, we used a total of seven different datasets, which cover many types of medical images. Because the datasets are different in the competitions, the number of images and the sizes of the images. Therefore, the pre-processing for the datasets is necessary to unify them for the models. The preprocess would be dissimilar for different datasets. It depends on image resolution as well as the ratio between positive and negative samples. Table 2 summarizes the detailed information of datasets used in our experiments. 

#### 3.1.1. Electron Microscopy (EM)

**Figure 5:**
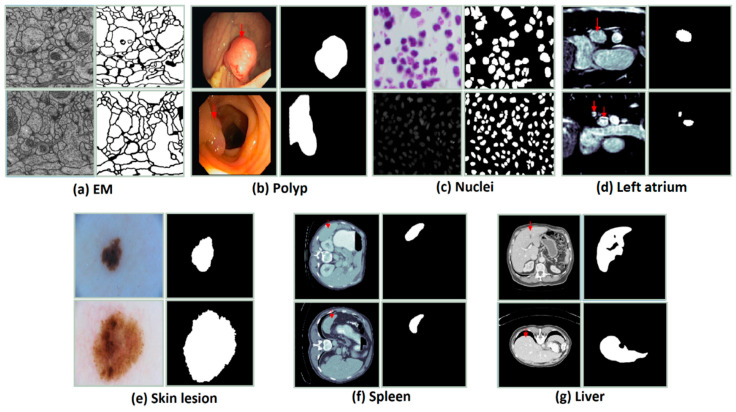
The examples of the datasets are used in the experiments. The first column shows the original images and the second one presents the ground truth.

The dataset is provided by a part of the IEEE International Symposium on Biomedical Imaging (ISBI) 2012 [47] for the challenge of segmentation of neuronal structures in EM stacks. The dataset is a set of 30 images with a size of 512 × 512 pixels from the electron microscopy images of the Drosophila first instar larva ventral nerve cord (VNC). Figure 5a shows an example of an image in the dataset. The image annotation is also provided. The white pixels indicate the cells while the black pixels present the membranes. The dataset was split into three parts: training part (22 images), validation (3 images), and testing (5 images). For training and testing, we applied the sliding window with the size of 128 × 128 and the overlap area of two adjacent windows is 64 × 128. Finally, the total number of images for training, validation, and testing are 1078, 147, and 245, respectively. The final evaluation results of our experiments are done based on the images with a size of 128 × 128.

#### 3.1.2. Polyp (CVC-ClinicDB)

The dataset is provided by the 2015 MICCAI sub-challenge on automatic polyp detection [48]. The dataset consists of 612 images (almost of them is a size of 384 × 288) are extracted from 25 different colonoscopy videos, shows several points of view of the polyp. The ground truth is the mask corresponding to the polyp region in the image. For training and testing, the images are resized to 224 × 224. The dataset is divided into two sets: a training set (489 images) and a testing set (123 images). In the training process, we split the training and validation data with a ratio of 80% and 20%.

#### 3.1.3. Nuclei

This dataset is supplied by the segmentation challenge of Data Science Bowl 2018 (DSB challenge 2018) [49]. The dataset includes 670 nuclei images, which almost is the size of 256 × 256 × 3, from different modalities: brightfield vs. fluorescence. Figure 5c presents the examples of the image in the dataset and the ground truth. The dataset is randomly split into a training set (423 images), a validation set (108 images), and a testing set (130 images). For both training and testing the model, the images with a size of 128 × 128 were used. We simply resized the original images to the desired size.

#### 3.1.4. Left Atrium

The dataset is provided by the Medical Segmentation Decathlon Challenge 2018 (MSD 2018) [50]. It consists of 20 MRI volumes for training and 10 volumes for testing. In our experiments, we only use the training part. There are 2271 slices with a size of 320 × 320 pixels. The dataset is divided into two parts: 15 volumes for training and validation (1702 slices, 20% used for validation), 5 volumes (569 slices) for testing. The Hounsfield unit window in the range of [500, 1500] is also applied to the slices. To reduce the computation time and the fraction between the positive class and negative class, the images were cropped to a size of 128 × 128.

#### 3.1.5. Skin Lesion

The dataset is supplied by the ISIC-2018 Challenge [51] and consists of 2594 high-resolution dermoscopy images. The size of the images in the dataset is pretty different. The images were re-scaled to a size of 224 × 224 to reduce the calculation time. The dataset is randomly split into a training set (1660 images), a validation set (415 images), and a testing set (519 images). Figure 5e presents the examples of the image in the dataset and the ground truth.

#### 3.1.6. Spleen

The dataset is provided by the Medical Segmentation Decathlon Challenge 2018 (MSD 2018) [50], which consists of 41 CT volumes for training and 20 CT volumes for testing. In our experiments, we only use the training part for evaluation that includes 3650 images (512 × 512 pixels). The dataset was randomly divided into three parts: 2920 slices for training, 584 slices for validation, and 730 slices for testing. The Hounsfield unit window in the range of [−200, 250] is also applied to the slices. To reduce the computation time and the fraction between the positive class and negative class, the images were cropped to a size of 224 × 224.

#### 3.1.7. Liver

The dataset is supplied by the 2017 LiTS challenge and includes 201 CT volumes. The ground truth is only accompanied by 131 CT volumes, thereby we only use this part in our experiments. The dataset was collected from different hospitals and institutions [52]. The goal of the LiTS challenge is the extraction of the liver and liver tumor but in our experiments, we only perform the liver segmentation. There is a total of 58,638 2D slices with a size of 512 × 512. The dataset was split into a test set (90 volumes), a validation set (11 volumes), and a test set (30 volumes). To reduce the computation time, we first cropped the image into a size of 448 × 448 then re-scaled to the size of 224 × 224. To exploit the z-information in the volumes, the previous slice and the next slice with the slice that is considered are combined. The Hounsfield unit window was also applied in the range of [−200, 250]. The ratio between the image without the liver and the image with the liver is high. To tackle this problem, two slices in three continuous slices that are without the liver were excluded. Finally, the total images used in our experiments are 22,109, 4494, and 7059 images for training, validation, and testing, respectively. The size of the images is 224 × 224 × 3.

### 3.2. Experiment Setting 

In this section, the details of the setting in our training process would be described. In our experiments, four network models were implemented: Unet, Unet++, T-Unet, and TMD-Unet. All the models were trained and tested on seven datasets. With the same application, we applied the same setting and training strategies for all the models. Table 3 summarizes the details of our setting for training all the datasets. 

The loss function plays an important goal in improving the performance of the models [53]. The problem of medical image segmentation is the data imbalance. To tackle this problem, the hybrid loss function was used. In our experiments, the loss function is a combination between the dice loss and the cross-entropy loss. Since the evaluation was performed on multiple datasets, applying the same loss function will result in inefficiency for some datasets. The combination of dice loss and cross-entropy will solve the data imbalance problem, which is the difference between the positive class and the negative class. For the segmentation challenges, the value is appreciated as the dice coefficient. In this study, hence, the dice loss and cross-entropy loss were chosen. Because of the difference between the ratio of positive class and negative class in the datasets, we used two types of cross-entropy loss that are the binary cross-entropy (BCE) and weighted cross-entropy (WCE). The BCE is used for the dataset without the imbalance classes while the WCE is used for the datasets that are imbalance classes. The formula of the hybrid loss is expressed by

(8) $$L_{total}=L_{Dice-loss}+L_{Cross-Entropy}$$

where *L_Cross-Entropy_* and *L_Dice-loss_* represent the cross-entropy loss and the dice loss, respectively. The WCE loss, BCE loss, and the dice loss are computed as:

(9) $$L_{WCE}=-\frac{1}{N}\sum_{i=1}^{N}\left(\left(1-w\right)y_{i}\log k_{i}+w\left(1-y_{i}\right)\log\left(1-k_{i}\right)\right)$$

(10) $$L_{BCE}=-\frac{1}{N}\sum_{i=1}^{N}\left(y_{i}\log k_{i}+\left(1-y_{i}\right)\log\left(1-k_{i}\right)\right)$$

(11) $$L_{Dice-loss}=1-\frac{2\sum_{i=1}^{N}\left(y_{i}k_{i}\right)+\delta }{\sum_{i=1}^{N}\left(y_{i}+k_{i}\right)+\delta }$$

where *y_i_* indicates the ground truth value of the *i* pixel and *k_i_* presents the predicted value of the *i* pixel. The *N* is the total number of the pixels, and the *w* denotes the weight of the foreground class, and the δ is the smooth value that prevents the problem of divide by zero.

All our experiments are deployed by Keras package and Tensorflow version 2.0.0 is a backend. To initialize the weights of the models, the he-normal distribution initializer that was proposed by He et al. [54] was used. An Adam optimizer is used for all models. The initial learning rate (ILR) value is set to 3 × 10^−4^ for all applications except the left atrium segmentation application, the ILR value is set to 1 × 10^−3^. The learning rate will be adjusted by a learning rate scheduler, according to the formula:

(12) $$lr=ILR\times \left(0.9^{E/10}\right)$$

where *lr* is the learning rate, *E* is the epoch numbers. To prevent over-fitting, a dropout rate of 0.2 is applied. To save the training time, an early-stopping mechanism was also applied when training the models. The experiments are conducted by a workstation with Intel Xeon Silver 4114 CPU, GRID Virtual GPU V100D-8Q, and 32 GB of RAM memory.

Due to the limitation of the number of images in datasets, the data augmentation techniques were applied for all applications except the liver segmentation. The data augmentation techniques are the same for all applications, including shearing, rotation, zoom, flip, and shift. The image data generator was used to create the training and validation data with the same random seed for all the network models. The details of the setting data generator were the shear range of 0.5, rotation range of 50 degrees, the zoom range of 0.2, the horizontal flip of true, width shift range of 0.2, height shift range of 0.2, and fill-mode is reflection.

## 4. Results

### 4.1. Evaluation Metrics

In this study, we used six metrics to evaluate the model performance: Dice coefficient (DSC), mean Intersection over Union (mIoU), Recall (RE), Precision (PR), Specificity (SP), and F1-score (F1). The expressions of the metrics are described as follow:

(13) $$DSC(Y,\hat{Y})=\frac{2\left|Y\cap \hat{Y}\right|}{\left|Y\right|+\left|\hat{Y}\right|}$$

(14) $$mIoU(Y,\hat{Y})=\frac{\left|Y\cap \hat{Y}\right|}{\left|Y\cup \hat{Y}\right|}$$

(15) $$PR=\frac{TP}{TP+FP}$$

(16) $$RE=\frac{TP}{TP+FN}$$

(17) $$SP=\frac{TN}{TN+FP}$$

(18) $$F1=2\times \frac{PR\times RE}{PR+RE}$$

where *Y* denotes the case of ground truth values, $\hat{Y}$ denotes the case of predicted values. The *TP*, *FP*, *TN*, and *FN* depict the case numbers of true positives, false positives, true negatives, and false negatives, respectively. For the evaluation metrics, the greater values indicate better efficiency, and the most valuable metrics to evaluate the performance of the model are DSC, mIoU, and F1.

### 4.2. Segmentation Results

This section shows the segmentation results on seven datasets. Table 4 compares the segmentation results of the Unet, Unet++, T-Unet, and TMD-Unet models in terms of all metrics used in our experiments. As seen in the table, for the EM segmentation, the evaluation results of the Unet model are the lowest except for the RE metric. Unet++ performs better than Unet on all metrics except the RE value. T-Unet outperforms the Unet but not as good as Unet++. Our final proposed model, TMD-Unet accomplished the best results on five crucial metrics that are DSC, mIoU, F1, PR, and SP. On the results sheet, T-Unet got the best results on the RE metrics (97.01%), TMD-Unet achieved outstanding results contrasted to other models. Comparison with traditional Unet, the evaluation values of TMD-Unet increased by 0.83%, 0.32%, 0.5%, 1.55%, and 3.63% respectively for DSC, F1, mIoU, PR, and SP. We also notice that the SP value is much smaller than the RE and PR metrics. The possible reason is the ratio of the positive class and the negative class is tiny, which leads to the case number of FP will be higher. Therefore, the SP value in the EM application is smaller than other metrics and in other applications.

For nuclei dataset, we can see that Unet achieved the best results on the RE metric (94.95%), but the remaining values are the worst. Unet++ obtained better results on two metrics PR and SP (93.94% and 98.53%). For the three valuable metrics DSC, mIoU, and F1, TMD-Unet achieved the best results (92.49%, 94.04%, and 86.08%). T-Unet model also performed better results than Unet and Unet++ on three crucial values. Although our models did not achieve higher RE and PR values, these values were more balanced than Unet and Unet++, which is the reason the DSC, F1, and mIoU metrics of T-Unet and TMD-Unet are better than Unet and Unet++.

The segmentation results of TMD-Unet on polyp segmentation application outperformed the remaining models on all evaluation metrics. Comparison with Unet, the improvement is 2.45%, 2.51%, 4.21%, 2.14%, 3.73%, and 0.43% for DSC, F1, mIoU, RE, PR, and SP, respectively. T-Unet model also surpassed Unet and Unet++. As shown in the table, we can see that Unet++ got the worst results of the three values: DSC, mIoU, and RE. We observe that the RE value obtained by Unet++ is the smallest (88.62%), this proves the ratio of TP/FN is also the lowest. Although the PR metric of Unet++ is better than that of Unet, it has no balance between RE and PR. Therefore, the evaluation metrics of Unet++ is lower than the other models.

The left atrium segmentation and skin lesion segmentation have obtained the same outcome scenario. Unet++ got the worst results on valuable metrics. TMD-Unet model performed the best results on the DSC, F1, mIoU, and RE. T-Unet obtained the best results on the remaining two metrics, PR and SP. For skin lesion dataset, TMD-Unet performed 4.12%, 4.14%, 6.44%, 6.27% higher than Unet and 4.43%, 3.13%, 36.5%, 3.73% higher than Unet++ corresponding to DSC, F1, mIoU, RE. For the left atrium dataset, the increments are 1.2%, 1.63%, 1.86%, and 3.37% in comparison with the Unet; 2.85%, 2.55%, 4.59%, and 0.43% contrasted to Unet++. The PR value achieved by Unet++ is the smallest, proving that the ratio of FP/TP is higher than that of other models. This is the reason other metrics of Unet++ are also lower. For TMD-Unet, the PR value is smaller than that of T-Unet; however, the RE value is superior to T-Unet, which leads to better results. 

The scenario of the spleen segmentation is similar to liver segmentation. TMD-Unet outperformed other models while Unet++ achieved the best results on the RE metric (95.94% for the spleen and 97.91% for the liver). Unet got the worst results on all the important metrics. For liver segmentation, T-Unet obtained worse results than Unet++. In contrast, for spleen extraction, T-Unet accomplished better results than Unet++. TMD-Unet showed remarkable improvement compared to Unet in terms of the DSC, F1, and mIoU on the spleen (5.13%, 5.29%, and 8.39%) and the liver (5.17%, 5.33%, and 9.06%) segmentation. Unet++ achieved the highest RE value. This shows that Unet++ is possible highly effective in identifying the positive class. Our model, TMD-Unet, obtained the highest result on PR value (96.40% for spleen, 95.71% for liver). This proves that TMD-Unet is possible more effective at recognizing the negative class. Furthermore, the RE value accomplished by TMD-Unet is also higher than Unet. Finally, the evaluation results of other metrics on TMD-Unet are better than other models.

**Figure 6:**
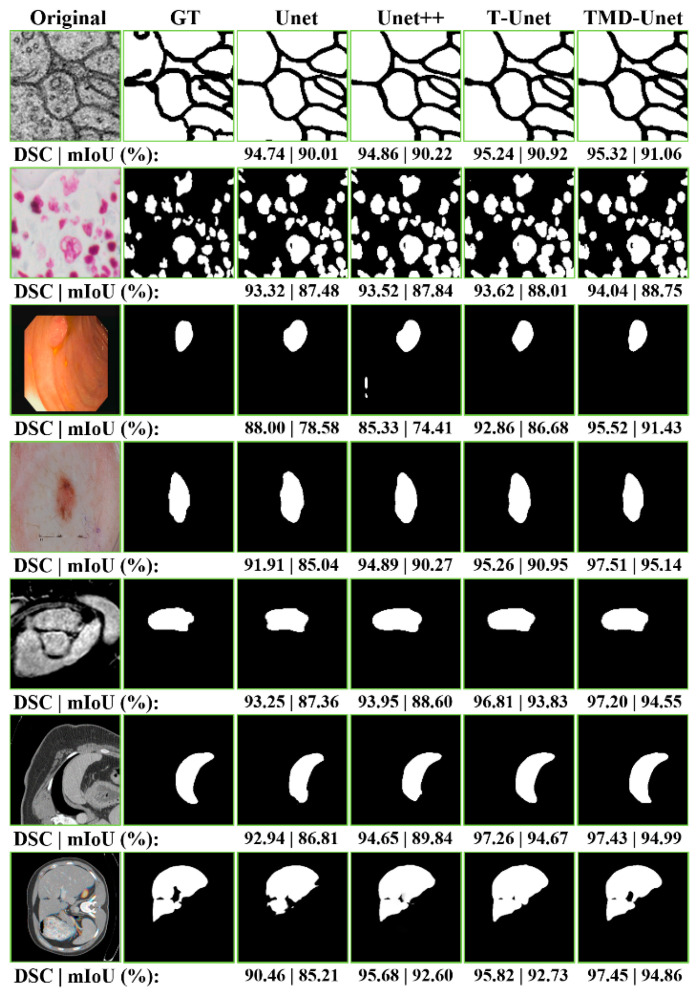
The examples of the segmentation results on the testing images of the dataset. The first column shows the original images while the second column indicates the ground truth (GT). From the third to the seventh column are the accomplished results by Unet, Unet++, T-Unet, and TMD-Unet, respectively. The segmented applications are listed in top-to-bottom rows: EM, nuclei, polyp, left atrium, skin lesion, spleen, and liver, respectively.

In Table 4, the qualitative evaluations are presented. To authenticate the quantitative evaluations, some examples from the segmentation results on the testing set of the datasets are presented in Figure 6. We observe that the extraction results of Unet model always contain many errors compared to the ground truth. Unet++ accomplished better results in comparison with Unet. For polyp segmentation, however, there are still many false positives cases. The proposed models achieve more definite results than Unet and Unet++. Figure 6 also shows the DSC and mIoU values for each result. Observing the segment results in the figure, we can see that the number of FP cases of Unet and Unet++ is much higher than that of T-Unet and TMD-Unet. The FP values mostly appear in the boundary area of the object, where the difference between the background and foreground is not much and not obvious. This demonstrates a significant efficiency improvement of the proposed model compared to Unet and Unet++.

### 4.3. Feature Map Visualization 

The exploiting all output features from the convolutional units in the encoder node enhanced the feature maps of the decoder nodes. Unet with only one output, the features from the encoder are insufficient, leading to poor results. For Unet++, the skip connection incorporated more features, included four sub-Unet models with different depths. However, the sub-Unet models are not deep enough. In our proposed model, although only 3 sub-Unet models are included, the depth is still guaranteed. The sub-models are behind, and the greater the number of filters at the nodes ensure the extraction of image features. In this section, we will illustrate the structure and output features from the layers of Unet, Unet++, T-Unet, and TMD-Unet models in detail.

**Figure 7:**
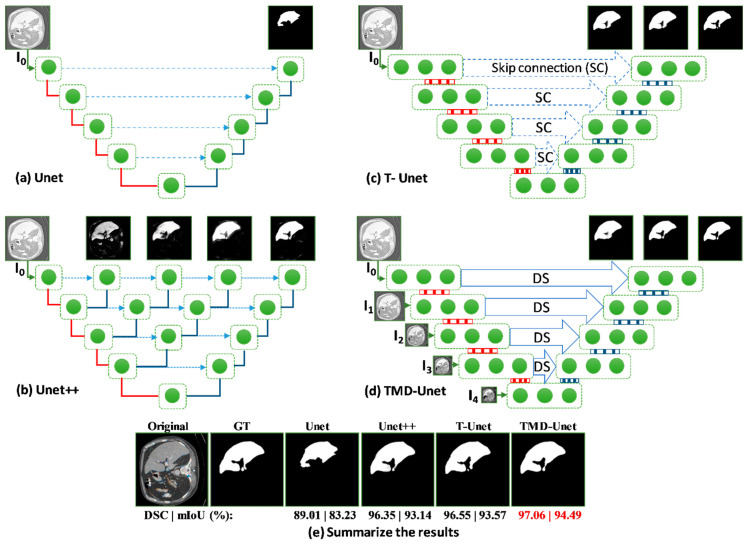
Visualization of the feature maps from the inputs and outputs on the models for the liver CT image. The (**a**–**d**) show the structure and output features of Unet, Unet++, T-Unet, and TMD-Unet, respectively. The (**e**) presents the final outputs and the metrics of all the models. The red lines indicate max-pooling while the blue lines are transposed convolution. The green dot denotes the convolution unit of the node.

Figure 7 shows the details of the architecture and illustrates the input and output feature maps of the models. As you can see in the figure, the output from Unet model loses a lot of information. For Unet++, the outputs from the first sub-models still have many false positives, the outputs are gradually improved for the following sub-model outputs. The possible reason is that for the first sub-models, the depth of the model is not enough, leading to limited results. The following sub-models have improved in depth, so the results are getting better. For T-Unet and TMD-Unet, the sub-models have the same depth but different in the number of filters. The results in the figure show that the first sub-models achieved better results than the first one of Unet++, the following models had significant enhancements. The integration of MSI and DS into TMD-Unet model helps to combine more features from the input side and better information from the encoder to provide to the decoder part. The results in Figure 7 also shows the better results of TMD-Unet in comparison with T-Unet.

### 4.4. Comparing with Recent Models

In this section, we compare the segmentation result of our models with some recent network models in terms of DSC, F1, and mIoU metrics. In addition to Unet and Unet++, the models used to comparison in this study include the Unet-based models (Double-Unet [39], R2U-Net [32], CU-Net [38], Multi-ResUnet [36], Cascade U-Resnet [10]), and others architecture networks (Deeplab V3+ [55], Generative Adversarial Network (GAN) [56]). Table 5 compares the proposed model results with the current models. We observe that our models outperformed other networks on most of the applications except for the skin lesion segmentation. The possible reason is that the Double-Unet was trained and tested with the larger size of the image (384 × 512), and the pre-processing was applied to the DeeplabV3+. For the spleen and atrium segmentation, our models achieved a better result than the fifth-ranked in the leader board of the Medical Segmentation Decathlon Challenge. 

## 5. Discussion

Unet architecture is still the most successful architecture on the medical image segmentation challenge. Almost all current models are based on Unet to apply to the problem of image segmentation. The most recent is the Unet++ model, an enhancement of the Unet model that has also been widely used as a background architecture for image processing applications. However, the limitation of generality is still a disadvantage of these network models. As presented in the experimental section, the Unet++ model outperformed Unet in several applications (EM, nuclei, spleen, and liver). However, in some other applications (polyp, skin lesion, left atrium), the results are the opposite, Unet++ performed worse than Unet. For our proposed model, evaluation results showed superiority over Unet and Unet++ across all applications.

The analytical results in Figure 7 have shown the effectiveness of the proposed model. The inefficiency of Unet is due to its simple architecture. For Unet++ model, the first sub-models are still not deep enough, so the segmentation results are still limited. Table 6 shows the parameter numbers for the Unet, the Unet++, T-Unet, and TMD-Unet. For T-Unet and TMD-Unet models, the parameter numbers of the sub-models are acceptable from the original sub-models. The downside of deep learning models is their generality. In this study, we proposed and evaluated the model on seven data sets. The results showed significant improvement in some datasets such as skin lesion, liver, and spleen. Using the DC in place of the standard convolution is also effective for images that include large objects. This is evident in the improvement of the spleen and liver application in comparison with other applications. The improvements in metrics DSC and mIoU contrasted to Unet were 5.13% and 8.39% respectively on the spleen and 5.17% and 9.06% on the liver, respectively (in Table 5). The incorporation of MSI and DS into the proposed model has also achieved performance improvement. Across all applications, the TMD-Unet consistently outperforms the T-Unet on the critical evaluation metrics.

The exploiting of feature maps has shown significant efficiency. The integration of MSI and DS has also obviously improved the performance of the model. Compared with Unet and Unet++, the proposed network size is quite similar (see Table 6), but the efficiency shows an improvement. That is also an advantage of the proposed model. However, the disadvantage of the model is that the number of parameters and computation time will dramatically increase as the filter numbers of convolutional units are growing. In this study, because of the computer capabilities, we did not accomplish a comparison when changing the number of filters for convolutional units on the proposed models.

## 6. Conclusions

In summary, we have proposed a new network model based on an improvement from the well-known network model, which is the Unet model. In the new architecture, the interconnections of the nodes were modified, and the intra-features were exploited more effectively. In addition, the multiple-input features in conjunction with DS connectivity also showed a significant improvement in terms of the segmentation results. The generality and applicability of the proposed model have been demonstrated in this study, specifically, our experiments performed on seven datasets with different numbers of samples and data types. The results showed the dominance of the proposed model across all datasets. However, the disadvantage of the network is that the network size and computation time dramatically increase if the filter numbers of convolutional units are growing. It is believed that our proposed model can be considered to segment other types of medical images such as Positron emission tomography (PET) or ultrasound.

## Figures and Tables

**Table 1:** The architecture of TMD-Unet.

Node	MSI	Encoder	DS	Decoder	Output
1	16 × (3 × 3 Conv)	[16 × (DC3 + ReLU)]^2^ + BN Max-pooling [16 × (DC2 + ReLU)]^2^ + BN Max-pooling [16 × (DC1 + ReLU)]^2^ + BN Max-pooling	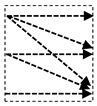	[16 × (DC3+ ReLU)]^2^ + BN Transposed Convolution [16 × (DC2+ ReLU)]^2^ + BN Transposed Convolution [16 × (DC1+ ReLU)]^2^ + BN Transposed Convolution	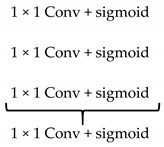
2	Max-pooling	[32 × (DC3 + ReLU)]^2^ + BN Max-pooling [32 × (DC2 + ReLU)]^2^ + BN Max-pooling [32 × (DC1 + ReLU)]^2^ + BN Max-pooling	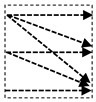	[32 × (DC3+ ReLU)]^2^ + BN Transposed Convolution [32 × (DC2+ ReLU)]^2^ + BN Transposed Convolution [32 × (DC1+ ReLU)]^2^ + BN Transposed Convolution	
3	Max-pooling	[64 × (DC3 + ReLU)]^2^ + BN Max-pooling [64 × (DC2 + ReLU)]^2^ + BN Max-pooling [64 × (DC1 + ReLU)]^2^ + BN Max-pooling	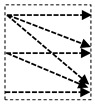	[64 × (DC3+ ReLU)]^2^ + BN Transposed Convolution [64 × (DC2+ ReLU)]^2^ + BN Transposed Convolution [64 × (DC1+ ReLU)]^2^ + BN Transposed Convolution	
4	Max-pooling	[128 × (DC3 + ReLU)]^2^ + BN Max-pooling [128 × (DC2 + ReLU)]^2^ + BN Max-pooling [128 × DC1 + ReLU)]^2^ + BN Max-pooling	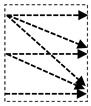	[128 × (DC3+ ReLU)]^2^ + BN Transposed Convolution [128 × (DC2+ ReLU)]^2^ + BN Transposed Convolution [128 × (DC1+ ReLU)]^2^ + BN Transposed Convolution	
Tr.	Max-pooling	[256 × (DC3 + ReLU)]^2^ + BN [256 × (DC2 + ReLU)]^2^ + BN [256 × (DC1 + ReLU)]^2^ + BN			

DC*x* denotes the dilated convolution with a dilation rate equal to *x.* The superscript number indicates the number of repetitions. ‘Tr.’ is the transition.

**Table 2:** The details of the datasets are used in our experiments.

App.	No. of Images	Size	Modality	Provider
EM	30	512 × 512	Microscopy	ISBI 2012 [47]
Polyp	612	384 × 288	Colonoscopy	MICCAI 2015 [48]
Nuclei	670	256 × 256	Mixed	DSB 2018 [49]
Left atrium	2271	320 × 320	MRI	MSD 2018 [50]
Skin lesion	2594	Variable	Dermoscopy	ISIC 2018 [51]
Spleen	3650	512 × 512	CT	MSD 2018 [50]
Liver	58,638	512 × 512	CT	LiTS 2017 [52]

**Table 3:** The details of learning setting of the models.

App.	Loss	ILR	No. of Epochs	Batch Size	Input Size	Data Aug.
EM	DC + BCE ^1^	3 × 10^−4^	200	16	128 × 128	Yes
Nuclei	DC + BCE	3 × 10^−4^	100	16	128 × 128	Yes
Polyp	DC + WCE ^2^	3 × 10^−4^	200	8	224 × 224	Yes
Skin lesion	DC + BCE	3 × 10^−4^	200	8	224 × 224	Yes
Left atrium	DC + WCE	1× 10^−3^	100	16	128 × 128	Yes
Spleen	DC + WCE	3 × 10^−4^	200	8	224 × 224	Yes
Liver	DC + WCE	3 × 10^−4^	100	8	224 × 224	No

^1^ Dice loss + binary cross-entropy; ^2^ Dice loss + weighted cross-entropy.

**Table 4:** The comparison of the segmentation results. All metrics are in (%). The bold denotes the best one.

Applications	Models	DSC	F1	mIoU	RE	PR	SP
**EM**	Unet	93.82	94.81	88.39	96.12	93.27	75.64
	Unet++	93.94	95.05	88.60	95.56	94.61	78.70
	T-Unet	93.86	95.10	88.46	**97.01**	93.28	77.41
	TMD-Unet	**94.11**	**95.13**	**88.89**	95.46	**94.82**	**79.27**
**Nuclei**	Unet	91.87	93.65	85.00	**94.95**	92.43	98.19
	Unet++	92.24	93.98	85.64	94.08	**93.94**	**98.53**
	T-Unet	92.26	93.98	85.68	94.42	93.61	98.44
	TMD-Unet	**92.49**	**94.04**	**86.08**	94.77	93.37	98.41
**Polyp**	Unet	90.20	90.43	82.20	90.11	90.03	98.98
	Unet++	88.87	89.77	80.27	88.62	91.11	99.15
	T-Unet	90.84	91.25	83.38	91.37	91.25	99.11
	TMD-Unet	**92.65**	**92.94**	**86.41**	**92.25**	**93.76**	**99.41**
**Skin lesion**	Unet	83.15	84.28	71.22	78.77	90.72	97.87
	Unet++	82.84	85.29	71.16	81.31	90.46	97.80
	T-Unet	85.77	87.32	75.16	81.94	**93.60**	**98.49**
	TMD-Unet	**87.27**	**88.42**	**77.66**	**85.04**	92.53	98.25
**Left atrium**	Unet	90.61	90.65	83.05	92.89	88.60	99.70
	Unet++	88.96	89.73	80.32	95.83	84.45	99.56
	T-Unet	91.67	92.07	84.71	94.50	**89.85**	**99.73**
	TMD-Unet	**91.81**	**92.28**	**84.91**	**96.26**	88.67	99.69
**Spleen**	Unet	90.28	90.30	82.93	89.66	91.40	99.80
	Unet++	95.00	95.42	90.49	**95.94**	94.92	99.88
	T-Unet	95.15	95.43	90.81	95.60	95.28	99.88
	TMD-Unet	**95.41**	**95.59**	**91.32**	94.85	**96.40**	**99.92**
**Liver**	Unet	91.26	91.31	84.07	89.21	93.79	99.62
	Unet++	94.75	95.19	91.08	**97.91**	92.70	99.51
	T-Unet	95.85	96.09	92.08	96.34	95.66	99.72
	TMD-Unet	**96.43**	**96.64**	**93.13**	97.62	**95.71**	**99.72**

**Table 5:** Comparison of the proposed models with popular models. The bold data denotes the best value.

**Nuclei**				**Polyp**				**Skin Lesion**			
**Models**	**DSC**	**F1**	**mIoU**	**Models**	**DSC**	**F1**	**mIoU**	**Models**	**DSC**	**F1**	**mIoU**
Unet	91.87	93.65	85.00	Unet	90.20	90.43	82.20	Unet	83.15	84.28	71.22
Unet++	92.24	93.98	85.64	Unet++	88.87	89.77	80.27	Unet++	82.84	85.29	71.16
DoubleU-Net [39]	91.33	76.52	84.07	GAN [56]	88.48	-	81.27	Deeplab V3+ [55]	87.70	-	80.30
R2U-Net [32]	92.15	-	-	DoubleU-Net [39]	92.39	89.91	86.11	DoubleU-Net [39]	**89.62**	**91.06**	**82.12**
T-Unet	92.26	93.98	85.68	T-Unet	90.84	91.25	83.38	T-Unet	85.77	87.32	75.16
TMD-Unet	**92.49**	**94.04**	**86.08**	TMD-Unet	**92.65**	**92.94**	**86.41**	TMD-Unet	87.27	88.42	77.66
**Liver**				**EM**				**Spleen**			
**Models**	**DSC**	**F1**	**mIoU**	**Models**	**DSC**	**F1**	**mIoU**	**Models**	**DSC**	**F1**	**mIoU**
Unet	91.26	91.31	84.07	Unet	93.82	94.81	88.39	Unet	90.28	90.30	82.93
Unet++	94.75	95.19	91.08	Unet++	93.94	95.05	88.60	Unet++	95.00	95.42	90.49
CU-Net [38]	89.40	-	-	MultiResUnet [36]	-	-	88.72	T-Unet	95.15	95.43	90.81
Cascade U-Resnet [10]	94.90	-	90.50	T-Unet	93.86	95.10	88.46	TMD-Unet	**95.41**	**95.59**	**91.32**
T-Unet	95.85	96.09	92.08	TMD-Unet	**94.11**	**95.13**	**88.89**				
TMD-Unet	**96.43**	**96.64**	**93.13**								

**Table 6:** Comparison of the parameter numbers on the sub-Unet models.

Models	Number of Parameters (Million)			
	Output 1	Output 2	Output 3	Output 4
Unet	7.765	-	-	-
Unet++	0.101	0.530	2.316	9.539
T-Unet	1.944	4.477	8.384	-
TMD-Unet	2.018	4.749	9.119	-

## Data Availability

All datasets used in this paper are publicly available.
